# High pulse pressure and metabolic syndrome are associated with proteinuria in young adult women

**DOI:** 10.1186/1471-2369-14-45

**Published:** 2013-02-21

**Authors:** Jwa-Kyung Kim, Young-Su Ju, Sung Jin Moon, Young Rim Song, Hyung Jik Kim, Sung Gyun Kim

**Affiliations:** 1 Department of Internal Medicine, Hallym University Sacred Heart Hospital, Kidney Research Institute, Hallym University College of Medicine, 896, Pyeongchon-dong, Anyang-si 431-070, Dongan-gu, Korea; 2 Department of Occupational and Environmental Medicine, Hallym University Sacred Heart Hospital, Anyang, Korea; 3 Myongji Hospital, Kwandong University College of Medicine, Seoul, Korea

## Abstract

**Background:**

Obesity and metabolic syndrome play causative roles in the increasing prevalence of proteinuria in the general population. However, in young adult women the clinical significance of incidentally discovered proteinuria and its association with metabolic syndrome are unclear. We investigated the prevalence and risk factors for proteinuria in this population.

**Methods:**

A total of 10,385 women aged 20 to 39 years who underwent health screenings were surveyed. Each patient was tested for proteinuria with a dipstick (−, ±, 1+, 2+, or 3+), and proteinuria was defined as 1+ or greater. Persistent proteinuria was established by confirming proteinuria in a subsequent test. Metabolic syndrome was defined in accordance with the updated National Cholesterol Education Program Adult Treatment Panel III criteria for Asia.

**Results:**

The mean age was 28.9 ± 5.5 years, and the prevalence of persistent proteinuria was 1.0%. Among these subjects with persistent proteinuria, obesity and metabolic syndrome were found in 10.4% and 5.2%, respectively. Metabolic syndrome, as well as its components of hypertension, hyperglycemia, central obesity, low high-density lipoprotein levels, and high triglyceride levels, was closely related to the presence of proteinuria. In addition, a wide pulse pressure of ≥40 mmHg was another independent risk factor for proteinuria [odds ratio (OR) 3.29, 95% confidence interval (CI) 1.03–11.91)]. This had an additive effect on metabolic syndrome in terms of predicting proteinuria. Even in subjects without metabolic syndrome, the influence of an increased pulse pressure was consistent (OR 2.75, 95% CI 1.03–8.61).

**Conclusions:**

Specific attention to proteinuria may be necessary in asymptomatic young women aged 20 to 39 years if they have metabolic syndrome or a wide pulse pressure.

## Background

Proteinuria is a common laboratory finding in the general population. Although it is transient and functional in most cases, persistent proteinuria may be an independent risk factor for adverse renal and cardiovascular outcomes [1-4]. In clinical practice, nevertheless, incidentally discovered proteinuria in young adults aged 20 to 39 years is often overlooked as benign proteinuria. In fact, the Third National Health and Nutrition Examination Survey (NHANES III) in the United States reported that the prevalence of overt proteinuria (urine albumin-to-creatinine ratio [UACR] ≥ 300 mg/L) in this age group was 0.5% in males and 0.9% in females, and the relative frequency of an abnormal UACR of ≥30 mg/L was 6.5% and 8.1%, respectively, the lowest among all age groups [5]. Therefore, the cost-effectiveness and benefit of screening for proteinuria in young individuals without symptoms or relevant associated diseases such as diabetes or hypertension is debatable.

Metabolic syndrome, which comprises central obesity, dyslipidemia, high blood pressure (BP), and impaired fasting glucose, is a known risk factor for proteinuria in the general population [6,7]. Recently, the increasing prevalence of obesity has affected the increase in metabolic syndrome and glomerulopathy [8]. However, most studies of the relationship between metabolic syndrome and proteinuria were conducted in subjects over 40 years of age [9,10], and comparatively little is known in younger population.

Especially in young women aged 20 to 39 years, clinical proteinuria could be a critical issue because unrecognized or ignored pre-pregnancy proteinuria could induce many complications during pregnancy, from urinary tract infection (UTI) to chronic kidney disease, and it remains central to the diagnosis of preeclampsia in hypertensive pregnancy [11]. In addition, previous investigators found that even mild intermittent proteinuria in asymptomatic young individuals is frequently associated with significant underlying renal pathology, emphasizing that it may not be a benign condition [12].

Therefore, we evaluated the association between metabolic syndrome and proteinuria in this young female population and investigated additional risk factors for proteinuria.

## Methods

### Subjects and health screenings parameters

A total of 10,724 young women aged 20–39 years who underwent health screenings at Hallym University Sacred Heart Hospital from January 2010 to December 2011 were enrolled. Among those, medical records were available for 10,472 subjects. To exclude the possibility of false-positive results, alkaline (pH ≥8, n = 39) or highly concentrated (specific gravity >1.025, n = 33) specimens were excluded. Individuals with pre-diagnosed CKD (n = 15) was also excluded. Thus, the data of 10,385 subjects were analyzed. This study was performed with the approval of the local Institutional Review Board.

The health screening examination included 1) an interview regarding current health status (diabetes, hypertension, alcohol, smoking, exercise, medication, marital status, and childbirth), 2) a physical examination - height, weight, waist circumference, body mass index (BMI) and blood pressure (BP) 3) blood and urine tests. Waist circumference was measured to the nearest 0.1 cm in a horizontal plane at the level of the midpoint between the iliac crest and the costal margin at the end of a normal expiration. The BMI was calculated as the individual’s weight (kg) divided by the square of the height (m), and obesity was defined as BMI ≥25 kg/m^2^. All blood samples were obtained in the fasting state. The serum levels of hemoglobin, glucose, total cholesterol (TC), high-density lipoprotein (HDL) cholesterol, triglycerides (TG), and creatinine were measured. Low-density lipoprotein (LDL) cholesterol was calculated using the following equation; LDL = TC – HDL – TG/5.0 (mg/dL). Glomerular filtration rate (GFR) was assessed using 2 estimating equations, Modification of Diet in Renal Disease (MDRD) Study equations and CKD Epidemiology Collaboration (CKD-EPI) equations [13]. Metabolic syndrome was defined in accordance with the updated National Cholesterol Education Program Adult Treatment Panel (NCEP-ATP) III criteria [14]. Particularly in our study, waist criteria for Asian people were used [15,16]. The presence of three or more of the following criteria constituted a diagnosis of metabolic syndrome: (i) waist circumference ≥80 cm; (ii) fasting triglyceride ≥150 mg/dL or medication use; (iii) HDL cholesterol < 50 mg/dL or medication use; (iv) BP ≥130/85 mmHg or antihypertensive medication use; and (v) fasting glucose ≥100 mg/dL or current medication.

### Proteinuria evaluation

Dipstick urinalysis was performed using spontaneously voided fresh urine that was analyzed within a few minutes after collection. Urinalysis was not performed in subjects who were menstruating or exhibiting symptoms of UTI or vaginal discharge. The results were interpreted by one physician and were scored as (−) when no staining was observed, (±) when weak staining was observed, and 1+, 2+, 3+, or 4+ when mild-to-strong staining was observed. Proteinuria was defined as 1+ or greater. However, the quantification of proteinuria was not performed in the routine screening test. For patients who had proteinuria at the initial test, follow-up test was recommended within 1 or 2 weeks. Persistent proteinuria was confirmed when repeat testing also revealed proteinuria ≥1+. For second test, proteinuria was quantified using protein-to-creatinine ratio (UPCR) and albumin-to-creatinine ratio (UACR). The clinical diagnosis of proteinuria was made by each physician based on previous history, clinical features, and laboratory findings regardless of whether a renal biopsy was performed or not.

### Statistical analysis

Statistical analyses were performed using SPSS version 18.0 (SPSS Inc., Chicago, IL, USA). All variables were expressed as the mean ± SD or median with ranges, unless otherwise indicated. Differences between two groups were analyzed by an independent *t*-test for continuous variables or the Fischer’s exact test for categorical data. The receiver operating characteristics (ROC) curve was constructed for evaluating the relationship between pulse pressure and proteinuria and the areas under the curve (AUC) were calculated. Multiple logistic regression analysis was performed to find significant determinants for proteinuria. In multivariate models, age, smoking, BMI, systolic BP, pulse pressure, cholesterol, presence of metabolic syndrome and eGFR_CKD-EPI_ were included. *P* < 0.05 was considered statistically significant.

## Results

### Baseline characteristics

**Figure 1:**
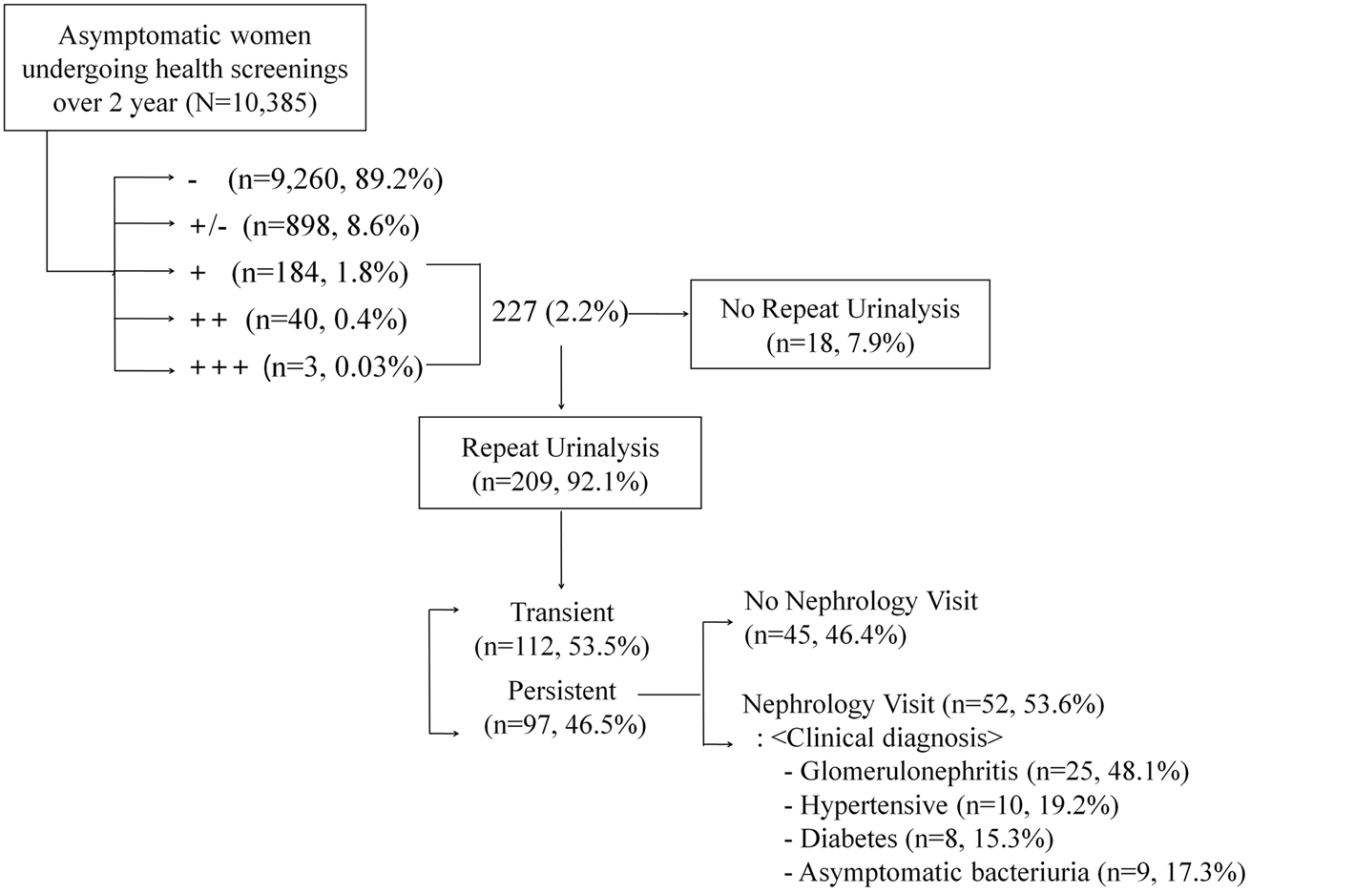
**Flow diagram of proteinuria evaluation.** The prevalence of persistent proteinuria was 1% in young women of general population.

The mean subject age was 28.9 ± 5.5 years, 6791 (65.3%) had a history of childbirth, and 1515 (14.6%) had been taking medication for dysmenorrhea. In the initial urinary dipstick test, 227 (2.2%) subjects had varying degrees of proteinuria: 1+ (n = 184, 1.8%), 2+ (n = 40, 0.4%), and 3+ (n = 3, 0.03%). Of these, a repeat test was performed in 209 subjects; 97 showed persistent proteinuria, but 112 had no proteinuria in the second urinalysis (Figure 1). Therefore, the prevalence of persistent proteinuria in young females aged 20 to 39 years was 1.0%. The median UPCR and UACR measured in repeat urinalysis were 0.57 and 0.45, respectively, in subjects with persistent proteinuria.

The baseline demographic and laboratory findings according to the presence of proteinuria are shown in Table 1. Persistent proteinuria was more prevalent in current or past smokers. In addition, subjects with proteinuria had a higher BMI, waist circumference, systolic BP, and wide pulse pressure. Baseline renal function, both the eGFR_CKD-EPI_ and eGFR_MDRD_, were significantly lower in subjects with proteinuria, too. In addition, serum levels of metabolic components, fasting glucose, TG, and HDL were significantly different between the two groups.

**Table 1:** Baseline characteristics of the study subjects

	Total*	**Persistent proteinuria**		
		**Negative**	**Positive**	**P**
Number, n (%)	10367	10270	97	-
Age (years)	28.9 ± 5.5	28.8 ± 5.5	29.3 ± 5.5	0.572
Smoker, n (%)				<0.001
Ex-smoker	95 (0.9)	87 (0.8)	8 (8.2)	
Current	102 (1.0)	93 (0.9)	9 (9.2)	
Height (cm)	161.5 ± 4.9	161.4 ± 4.9	162.4 ± 5.1	0.243
Weight (kg)	54.5 ± 8.5	54.0 ± 7.9	60.0 ± 12.4	<0.001
BMI (kg/m^2^)	20.9 ± 3.2	20.7 ± 2.9	23.2 ± 4.8	<0.001
Waist circumference (cm)	69.6 ± 7.3	69.3 ± 6.6	74.2 ± 11.6	<0.001
Systolic BP (mmHg)	107.0 ± 11.4	105.0 ± 10.9	118.1 ± 12.4	<0.001
Diastolic BP (mmHg)	66.7 ± 8.9	66.7 ± 9.0	66.3 ± 8.2	0.765
Pulse pressure (mmHg)	43.3 ± 9.3	38.4 ± 8.7	51.8 ± 11.1	<0.001
Hemoglobin (g/dL)	12.7 ± 0.9	12.7 ± 0.9	12.8 ± 1.2	0.552
Fasting glucose (mg/dL)	86.5 ± 13.3	85.7 ± 10.8	95.6 ± 28.5	<0.001
Serum creatinine (mg/dL)	0.66 ± 0.10	0.64 ± 0.09	0.70 ± 0.15	0.013
Estimated GFR				
CKD-EPI (mL/min/1.73 m^2^)	117.6 ± 10.8	117.7 ± 10.7	111.5 ± 17.1	<0.001
MDRD Study (mL/min/1.73 m^2^)	110.9 ± 33.1	111.0 ± 33.1	101.3 ± 23.2	0.048
Total cholesterol (mg/dL)	178.8 ± 29.4	177.8 ± 29.0	189.1 ± 31.7	0.015
HDL cholesterol (mg/dL)	67.6 ± 18.3	68.2 ± 18.5	62.2 ± 15.8	0.040
LDL cholesterol (mg/dL)	95.7 ± 27.3	94.7 ± 26.8	106.9 ± 30.7	0.006
Triglyceride (mg/dL)	78.0 ± 36.8	75.5 ± 35.0	104.7 ± 45.1	<0.001
UACR (mg/g)	-	-	0.45 (0.26-2.6)	-
UPCR (mg/g)	-	-	0.57 (0.31-3.6)	-
Blood on dipstick test, n (%)				0.015
None	8558 (82.6%)	8495 (82.7%)	63 (64.9%)	
Trace	1540 (14.8%)	1534 (14.9%)	6 (6.3%)	
Positive	269 (2.5%)	241 (2.3%)	28 (28.8%)	

*Total: All screening subjects (n = 10,385) – subjects without repeat urinalysis (n = 18).

Abbreviations: BMI, body mass index; BP, blood pressure; GFR, glomerular filtration rate; CKD-EPI, CKD-Epidemiology Collaboration; MDRD Study equation, Modification of Diet in Renal Disease Study equation; HDL, high-density lipoprotein; LDL, low-density lipoprotein; UACR, urine albumin-to-creatinine ratio; UPCR, urine protein-to-creatinine ratio.

### Prevalence of proteinuria and risk factor analysis

**Figure 2:**
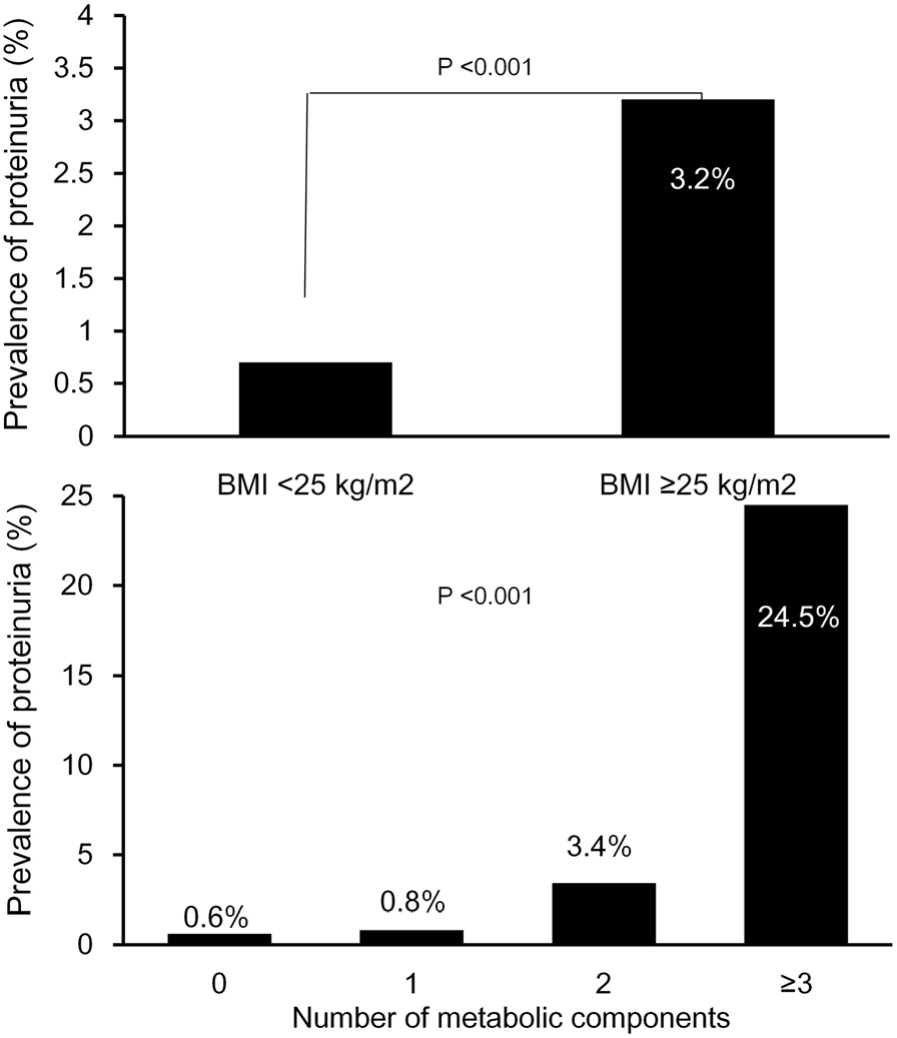
**The prevalence of proteinuria in subjects with obesity and metabolic syndrome.** The prevalence of proteinuria was significantly higher in obese patients, and the prevalence increased significantly as the number of metabolic components increased.

**Figure 3:**
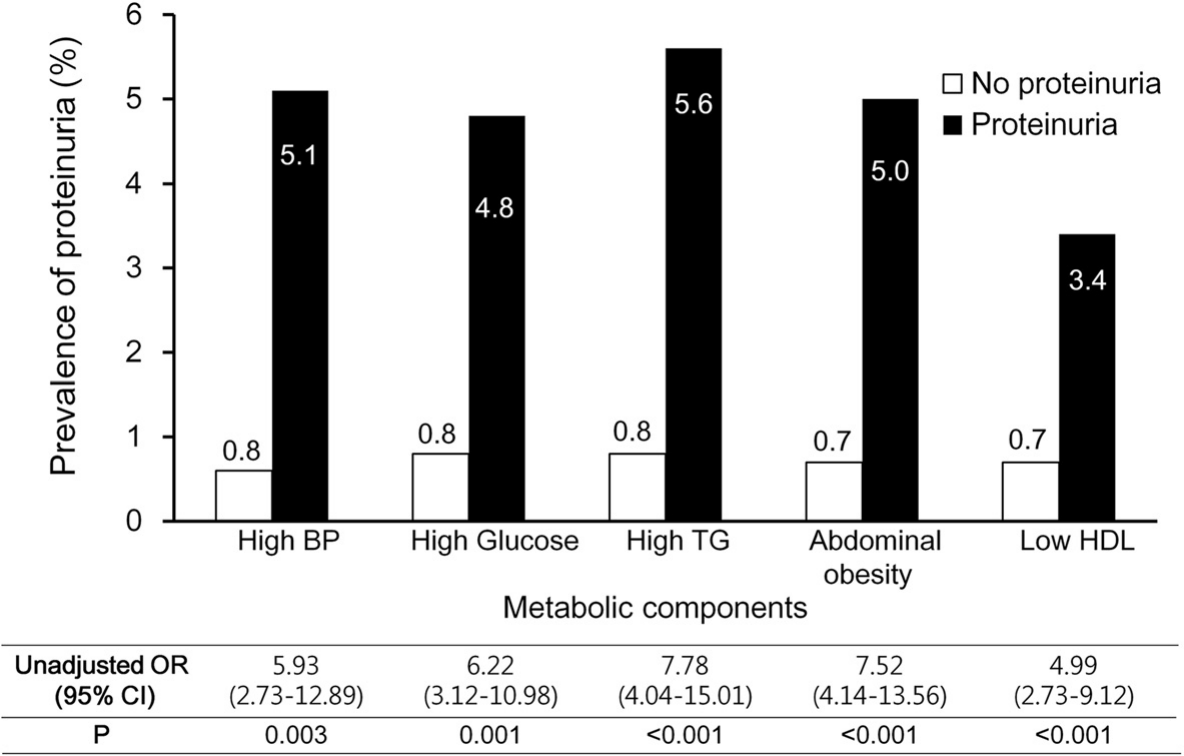
**Unadjusted odds ratio for proteinuria according to each metabolic component.** Significantly higher rates of proteinuria were observed across all categories of metabolic components.

Among the young women in this study, the prevalence of obesity and metabolic syndrome were 10.4% and 5.2%, respectively. The prevalence of proteinuria was higher in subjects with a BMI ≥25 kg/m^2^ compared with those with a BMI <25 kg/m^2^ (3.2% *vs*. 0.7%, respectively; *p* < 0.001) (Figure 2, upper). And proteinuria was detected more frequently in subjects with metabolic syndrome than in those without (24.5% *vs*. 0.7%, respectively; *p* < 0.001), and the prevalence increased significantly as the number of metabolic components increased (Figure 2, lower). In Figure 3, the unadjusted odds ratio (OR) for proteinuria according to each metabolic component is shown. Significantly higher rates of proteinuria were observed across all categories of metabolic components.

**Figure 4:**
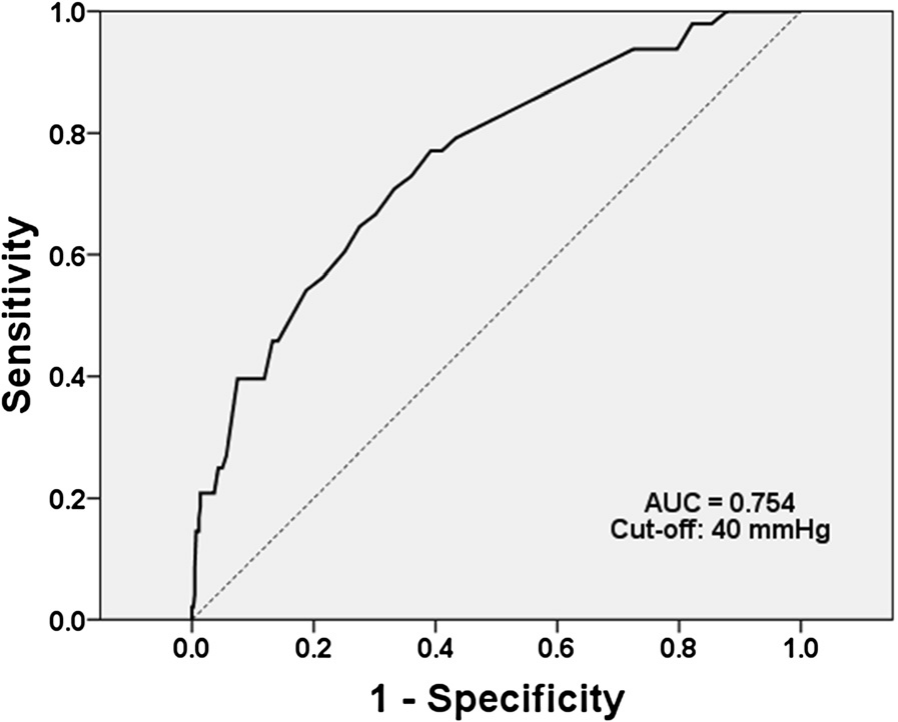
**ROC curve.** Pulse pressure of 40 mmHg maximizes the prediction of proteinuria (sensitivity of 91.2% and specificity of 84.4%). Area under the curve = 0.754.

**Figure 5:**
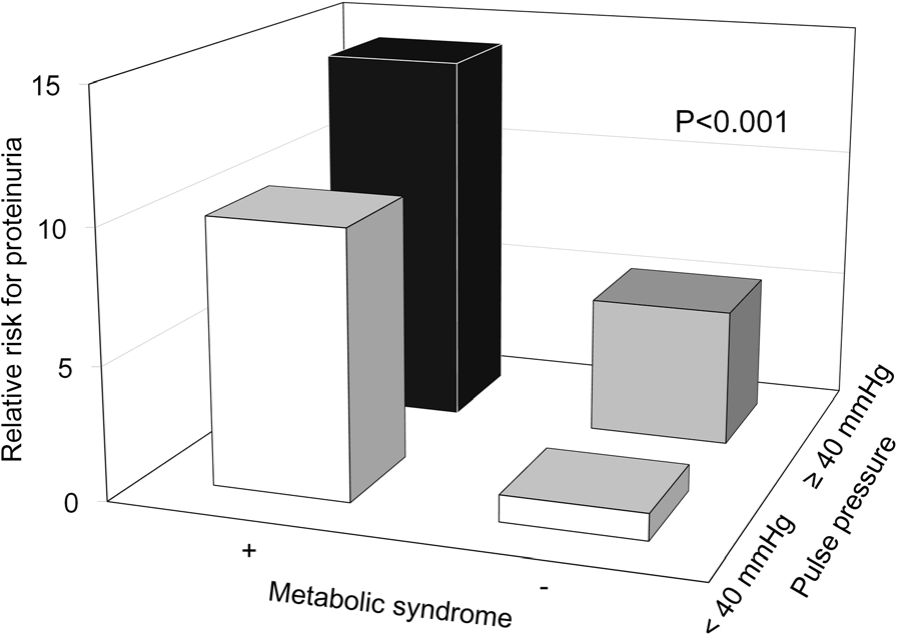
Subjects with both metabolic syndrome and elevated pulse pressure showed the highest relative risk for proteinuria in the general young population.

Furthermore, an increased pulse pressure was another important risk factor for proteinuria in these subjects. A 1-mmHg increase in pulse pressure was associated with a 10% increased risk of having proteinuria (OR 1.10, 95% CI 1.067–1.137, *p* < 0.001), and the ROC curve analysis showed a close relationship between pulse pressure and proteinuria (Figure 4). The area under the ROC curve was 0.754, and with a cut-off value of 40 mmHg, the sensitivity and specificity were 0.91 and 0.84, respectively. When subjects were stratified into four groups according to the presence of metabolic syndrome and pulse pressure (≥40 or <40 mmHg), the risk of proteinuria was highest in those with both metabolic syndrome and high pulse pressure (relative risk, 13.03) (Figure 5). Table 2 shows the clinical factors associated with proteinuria in this young female population. Smoking, a BMI of ≥25, systolic BP of ≥110 mmHg, pulse pressure of ≥40 mmHg, and the presence of metabolic syndrome were associated with proteinuria in the univariate analysis. In the multivariate analysis, only a pulse pressure ≥40 mmHg (OR 3.29, 95% CI 1.03–11.91) and metabolic syndrome (OR 7.77, 95% CI 3.27–18.44) were significant determinants of proteinuria.

**Table 2:** Univariate and multivariate analysis for prediction of proteinuria

	**Univariate analysis**		Multivariate analysis*		**Multivariate analysis†**	
	**OR (95% CI)**	**P**	**OR (95% CI)**	**P**	**OR (95% CI)**	**P**
**Total patients**						
Smoking	1.33 (1.06-6.83)	0.002	1.12 (0.78-2.59)	0.191	1.11 (0.59-1.66)	0.397
BMI ≥ 25	4.34 (2.05-9.18)	<0.001	1.61 (0.62-4.20)	0.262	1.53 (0.65-3.60)	0.321
Systolic BP ≥ 110 mmHg	2.39 (1.20-4.76)	0.013	1.69 (0.83-3.46)	0.145	1.64 (0.80-3.36)	0.174
Pulse pressure ≥ 40 mmHg	5.01 (1.53-16.58)	0.008	3.89 (1.08-10.36)	0.026	3.29 (1.03-11.91)	0.043
Metabolic syndrome	10.65 (5.10-27.96)	<0.001	7.99 (3.43-18.60)	<0.001	7.77 (3.27-18.44)	<0.001
**Without metabolic syndrome**						
Smoking	1.26 (0.58-3.66)	0.216	1.04 (0.54-2.35)	0.612	1.05 (0.54-3.05)	0.619
BMI ≥ 25	1.50 (0.43-5.23)	0.525	1.20 (0.36-4.02)	0.770	1.19 (0.35-4.01)	0.777
Systolic BP ≥ 110 mmHg	2.07 (0.95-4.50)	0.085	1.63 (0.73-3.62)	0.230	1.49 (0.74-3.64)	0.225
Pulse pressure ≥ 40 mmHg	3.68 (1.10-12.30)	0.032	2.89 (1.04-9.64)	0.038	2.75 (1.03-8.61)	0.041

* adjusted for age, smoking, BMI, systolic BP, pulse pressure, cholesterol, and presence of metabolic syndrome.

† adjusted for age, smoking, BMI, systolic BP, pulse pressure, cholesterol, presence of metabolic syndrome and eGFR_CKD-EPI_.

In the subgroup of subjects without metabolic syndrome, the prevalence of proteinuria was 0.7%. Smoking, a BMI ≥25, and a systolic BP ≥110 mmHg did not influence the presence of proteinuria. However, the effect of a wide pulse pressure was consistent even in subjects without metabolic syndrome (OR 2.75, 95% CI 1.03–8.61).

## Discussion

In the present study, we evaluated the prevalence of, and factors associated with, proteinuria in the general population, especially young women aged 20 to 39 years. We found that 1.0% of this population had persistent proteinuria, and metabolic syndrome as well as its components of hypertension, hyperglycemia, central obesity, low HDL levels, and high TG levels was closely related to the presence of proteinuria. In addition, a wide pulse pressure ≥40 mmHg was another important risk factor for proteinuria. The effect of a wide pulse pressure was independent of other clinical factors, including metabolic components. Even in subjects without metabolic syndrome, the influence of a wide pulse pressure on proteinuria was consistent. Therefore, specific attention may be necessary in asymptomatic young women if they have metabolic syndrome or a wide pulse pressure. To our knowledge, this is the first report of risk factors for proteinuria in young adult women.

In clinical practice, incidentally discovered proteinuria in young healthy adults is often ignored or overlooked. Indeed, according to a review article published two decades ago, proteinuria screening in healthy, asymptomatic adults was not recommended because less than 1.5% of subjects with positive dipstick findings had significant disease. However, they mentioned that proteinuria screening could detect many cases of potentially significant disease, such as mild glomerulonephritis or renal impairment [17]. Moreover, Muth *et al*. reported that proteinuria, even intermittent proteinuria, might not be a benign condition. They biopsied 51 young asymptomatic patients with intermittent proteinuria and normal renal function (age, 16–34 years) and found that approximately two-thirds of patients had pathologic evidence of renal disease [12]. As these data show, proteinuria in young adults may not be as benign as previously thought. Nevertheless, the recommendation of regular screening for proteinuria in all subjects in the general young population seems unreasonable because it is cost-ineffective. Therefore, early identification of risk factors for persistent proteinuria is important.

According to our data, the prevalence of incidentally discovered proteinuria was 2.2%, and half of those subjects had persistent proteinuria. Thus, the prevalence of persistent proteinuria was 1.0%, similar to previous reports [5]. Metabolic syndrome and its components were closely associated with persistent proteinuria. Metabolic syndrome, a clustering of various metabolic derangements, is a well-established independent predictor of cardiovascular morbidity and mortality in the general population [6,8,9,18,19]. In addition, a close relationship between metabolic syndrome and the development of proteinuria has been reported. Lucove *et al*. reported a 1.26-fold increased risk for the development of proteinuria among American Indians with metabolic syndrome [9]. Tozawa *et al*. also reported the effect of metabolic syndrome in a Japanese population (relative risk 2.09) [3]. Moreover, similar findings were observed in the general Korean population (OR 2.30). However, these prior studies included subjects over 40 years of age, and their significance in the young population is unknown. According to our data, in young subjects aged 20 to 39 years, metabolic syndrome was associated with an eightfold increased risk of persistent proteinuria. Several mechanisms, including insulin resistance, chronic inflammation, and lipotoxicity, have been proposed to explain the diverse renal effects of metabolic syndrome [20]. Various adipocytokines also play important roles in renal damage by inducing sympathetic overactivity, systemic inflammation, and oxidative stress [21]. Considering that metabolic syndrome is a modifiable risk factor, early detection and treatment of metabolic syndrome would be a cost-effective strategy to decrease the prevalence of proteinuria as well as chronic kidney disease and end-stage renal disease in the general population.

Another important finding of our study is that subjects with persistent proteinuria had a higher systolic BP and wider pulse pressure than those without persistent proteinuria. In particular, the pulse pressure was closely associated with proteinuria, and its effect was independent to other clinical factors. Generally in postmenopausal females, the loss of estrogenic action on the arterial wall appears to play a specific deleterious role by increasing arterial stiffness and pulse pressure [22,23]. Therefore, cardiovascular complications are more common in males and postmenopausal females than in premenopausal females. However, in this study, we found a harmful effect of an increased pulse pressure in young healthy women aged 20 to 39 years; therefore, our findings could emphasize the importance of pulse pressure on proteinuria independent to other risk factors such as aging process or hormonal changes. Our results are consistent with previous longitudinal data suggesting that pulse pressure is a risk factor for increased albuminuria in general or hypertensive atherosclerotic populations [24,25]. The close association between an increased pulse pressure and proteinuria could be explained by the fact that a wide pulse pressure induces endothelial dysfunction and barotrauma of the renal arteries [26-29]. However, due to the limitation of the present cross-sectional design, the causal relationship between proteinuria and wide pulse pressure among this young population without vascular risk factors could not be exactly identified. Moreover, a recent large-scale Japanese study reported that close associations between high pulse pressures and proteinuria could be established only in diabetic patients, not among subjects with prediabetes or normal glucose tolerance [30]. Therefore, a further well-designed study is needed to elucidate the pathophysiological link between pulse pressure and proteinuria in the young adult population.

The association between an increased pulse pressure and metabolic syndrome is also unclear; however, several studies investigated the relationship between pulse pressure and metabolic syndrome. Ferreira *et al*. reported that young individuals with metabolic syndrome have increased arterial stiffness because of poor cardiopulmonary fitness and high subcutaneous trunk fat [31]. The researchers suggested increased arterial stiffness as a risk factor for cardiovascular disease in subjects with metabolic syndrome. However, there is also a contrary opinion. According to a study by Mannucci *et al*., the close association between high pulse pressure and metabolic syndrome disappeared after adjustment for age and mean BP [32]. In our study, pulse pressure was closely associated with waist circumference, BMI, and TG levels, but not with HDL and glucose levels. Therefore, additional future data on this relationship are needed, too.

This study has several weaknesses. First, although the medical chart of each patient was thoroughly reviewed, it is impossible to review the precise history of past medical conditions, especially those affecting proteinuria, in all patients. Because the survey was a self-reported questionnaire, patients who were reluctant to report their medical conditions or drug history may not have provided completely accurate information. Moreover, we could not check several laboratory parameters that are known to be associated with proteinuria, such as the uric acid or C-reactive protein levels, because of the limitation of the health screening data. Second, of those with persistent proteinuria, only 53.6% were referred to a nephrologist and renal biopsy was performed only in a minority of patients; therefore, we were unable to identify the exact pathological diagnosis of persistent proteinuria.

## Conclusions

Of the young women aged 20 to 39 years in this study, 1.0% exhibited persistent proteinuria. Metabolic syndrome and its components of hypertension, hyperglycemia, central obesity, low HDL levels, and high TG levels were closely related to the presence of proteinuria. In addition, an increased pulse pressure may be an important independent risk factor for proteinuria; indeed, it exhibited an additive effect with metabolic syndrome for prediction of proteinuria. Specific attention to proteinuria may be necessary in asymptomatic young women aged 20 to 39 years if they have metabolic syndrome or a wide pulse pressure.

## Competing interests

The authors declare that they have no competing interests.

## Authors’ contributions

J-KK: Data analysis and writing up. Y-SJ: Data recruitment and analysis. SJM: Writing up and modification. YRS: Data analysis and statistical advisory. HJK: Study design determination. SGK: Research initiative and study design determination. All authors read and approved the final manuscript.

## Pre-publication history

The pre-publication history for this paper can be accessed here:

http://www.biomedcentral.com/1471-2369/14/45/prepub
